# Correction: Large-scale synthesis of ultrafine Fe_3_C nanoparticles embedded in mesoporous carbon nanosheets for high-rate lithium storage

**DOI:** 10.1039/d2ra90031a

**Published:** 2022-03-31

**Authors:** Ying Yu, Xuanli Wang, Hongkun Zhang, Zhiqin Cao, Haoyang Wu, Baorui Jia, Jun Jun Yang, Xuanhui Qu, Mingli Qin

**Affiliations:** Institute for Advanced Materials and Technology, University of Science and Technology Beijing Beijing 100083 China wuhaoyang@ustb.edu.cn qinml@mater.ustb.edu.cn; Inner Mongolia Key Laboratory of Advanced Ceramics and Device, School of Materials and Metallurgy, Inner Mongolia University of Science and Technology Baotou 014010 China; China United Test & Certification Co., Ltd Beijing 101407 China zhk@cutc.net; GRINM Group Corporation Limited Beijing 100088 China; College of Vanadium and Titanium, Panzhihua University Panzhihua 617000 China; Beijing Advanced Innovation Center of Materials Genome Engineering, University of Science and Technology Beijing Beijing 100083 China

## Abstract

Correction for ‘Large-scale synthesis of ultrafine Fe_3_C nanoparticles embedded in mesoporous carbon nanosheets for high-rate lithium storage’ by Ying Yu *et al.*, *RSC Adv.*, 2022, **12**, 6508–6514, DOI: 10.1039/d1ra08516f.

The authors regret that the affiliation for co-author Xuanli Wang was incorrectly shown in the original manuscript. The corrected list of affiliations is as shown here.

The Royal Society of Chemistry apologises for these errors and any consequent inconvenience to authors and readers.

## Supplementary Material

